# LongSAGE profiling of nine human embryonic stem cell lines

**DOI:** 10.1186/gb-2007-8-6-r113

**Published:** 2007-06-14

**Authors:** Martin Hirst, Allen Delaney, Sean A Rogers, Angelique Schnerch, Deryck R Persaud, Michael D O'Connor, Thomas Zeng, Michelle Moksa, Keith Fichter, Diana Mah, Anne Go, Ryan D Morin, Agnes Baross, Yongjun Zhao, Jaswinder Khattra, Anna-Liisa Prabhu, Pawan Pandoh, Helen McDonald, Jennifer Asano, Noreen Dhalla, Kevin Ma, Stephanie Lee, Adrian Ally, Neil Chahal, Stephanie Menzies, Asim Siddiqui, Robert Holt, Steven Jones, Daniela S Gerhard, James A Thomson, Connie J Eaves, Marco A Marra

**Affiliations:** 1 Genome Sciences Centre, British Columbia Cancer Agency, Vancouver, British Columbia, Canada, V5Z 1L3; 2 Terry Fox Laboratory, British Columbia Cancer Agency, Vancouver, British Columbia, Canada, V5Z 1L3; 3 National Cancer Institute, National Institutes of Health, Bethesda, Maryland 20892, USA; 4 Wisconsin National Primate Research Centre and Department of Anatomy, School of Medicine, University of Wisconsin, Madison, Wisconsin 53715, USA

## Abstract

To facilitate discovery of novel human embryonic stem cell (ESC) transcripts, we generated 2.5 million LongSAGE tags from 9 human ESC lines. Analysis of this data revealed that ESCs express proportionately more RNA binding proteins compared with terminally differentiated cells, and identified novel ESC transcripts, at least one of which may represent a marker of the pluripotent state.

## Abstract 2

Analysis of a 2.6 million longSAGE sequence tag resource generated from nine human embryonic stem cell lines reveals an enrichment of RNA binding proteins and novel ES-specific transcripts.

## Background

Embryonic stem cells (ESCs) can be derived from the inner cell mass of blastocysts and are defined by their ability to be propagated indefinitely as undifferentiated cells with the potential, upon appropriate stimulation, to generate cell types representing all three embryonic germ layers [1]. Since the first reported isolation of human cells with these properties [2], the derivation of more than 150 such lines has been described. This large collection of human ESC lines provides opportunities for understanding the earliest stages of human embryo and tissue development, as well as for elucidating the mechanisms that can permanently maintain pluripotency. Studies of mouse ESCs have defined a number of genes that appear to play key roles in this process, including those encoding Oct4 [3], Nanog [4,5], Sox2 [6], FoxD3 [7] and fibroblast growth factor-4 [8,9]. Comparisons of mouse and human ESCs have also revealed a number of conserved signaling pathways, including those involving JAK/STAT, transforming growth factor-β and fibroblast growth factor [10-12]. However, cross-species analysis of microarray data [13,14] and expressed sequence tag (EST) resources [15-18] suggest that additional molecular regulators of ESC self-renewal may exist and that likely candidates are heterochronic genes, microRNAs, genes involved in telomeric regulation and polycomb group repressors [14].

Microarray-based approaches have been used to define the transcriptomes of numerous human ESC lines, including BG01, BG02, WA01, WA07, WA09, WA13, WA14, TE06, UC01 and UC06 [19-22]. These studies provide a rich resource for cell line comparisons; however, incomplete annotation of the genome and inherent biases in the microarray technology limit interpretation to well characterized, abundantly expressed transcripts [23-25]. A number of DNA sequence-based approaches have also been used to study the human ESC transcriptome, including EST analysis [17], serial analysis of gene expression (SAGE) [15] and massively parallel signature sequencing (MPSS) [16,18]. Comparisons of these datasets have been used to search for genes that might be required for maintenance of pluripotency [13,15,16,22] but, interestingly, exhibit limited overlap between datasets, in some cases as low as 1% [26-28], possibly because of the different technologies employed in different studies [23]. The fact that a large proportion of transcripts expressed in ESCs do not correspond to annotated genes has further confounded the yields of such comparisons [14]. To generate a transcript discovery resource complementary to previous work, we undertook a large scale gene expression analysis of nine different human ESC lines, maintained as undifferentiated cells, using the long serial analysis of gene expression (LongSAGE [29]) approach.

## Results and discussion

### Digital gene expression profiling of nine human ESC lines reveals an enrichment of RNA binding proteins

LongSAGE libraries were constructed using total RNA purified from nine different human ESC lines cultured as undifferentiated cells by serial passaging on mouse embryonic fibroblast (MEF) feeder layers [30] (Table 1). To enable detection of the majority of the moderately to abundantly expressed transcripts, we sequenced most libraries to a depth of approximately 200,000 tags. However, in one case (the library prepared from WA09 cells), we generated 468,252 tags. To ensure that tags included in the libraries were not contaminated with transcripts expressed from the MEF feeder layers, all tags matching the mouse reference genome sequence were excluded from further analysis (Additional data file 1). SAGE libraries were analyzed individually, and also as an electronically pooled 'meta-library' containing 2.5 million tags representing 379,645 different tag sequences. Of these, 73% were observed only once ('singletons'). Our previous experience indicated that singletons are enriched for experimental artifacts (sequencing errors, reverse transcriptase artifacts, and so on) as well as rare transcripts [30]. To reduce the artifacts, we assigned confidence values to each tag sequence and selected for analysis only high quality tags as described [30]. This filtering reduced the total number of different tag sequences to 268,515 (Additional data file 2). Of these, 40% of the singletons and 87% of the non-singletons could be mapped to publicly available gene expression resources.

**Table 1:** Human embryonic stem cell lines profiled in this study

Provider's code	NIH code	Library identifier	Total no. of tags	Passage	Gender	Feeder line	Growth medium	bFGF-2 concentration (ng/ml)
H7	WA07	SHE13	272,470	22	Female	Mouse embryonic fibroblasts (CF-1)	DMEM:F12	4
H9	WA09	SHES2	468,040	38	Female	Mouse embryonic fibroblasts (CF-1)	DMEM:F12	4
H14	WA14	SHE14	212,211	22	Male	Mouse embryonic fibroblasts (CF-1)	DMEM:F12	4
H13	WA13	SHE15	221,117	22	Male	Mouse embryonic fibroblasts (CF-1)	DMEM:F12	4
HES-3	ES03	SHE10	206,292	16	Female	Mouse embryonic fibroblasts (B-81)	DMEM:F12	4
HES-4	ES04	SHE11	209,245	36	Male	Mouse embryonic fibroblasts (B-81)	DMEM:F12	4
UC06	HSF-6	SHES9	189,714	50	Female	Mouse embryonic fibroblasts (CF-1)	DMEM	10
H1	WA01	SHE16	218,214	54	Male	None: matrigel	DMEM:F12*	4
H1	WA01	SHE17	276,302	31	Male	Mouse embryonic fibroblasts (CF-1)	DMEM:F12	4
hESBGN-01	BG01	SHE19	201,699	20	Male	Mouse embryonic fibroblasts	DMEM:F12	4

*Mouse embryonic fibroblast conditioned media. bFGF-2, basic fibroblast growth factor.

**Figure 1:**
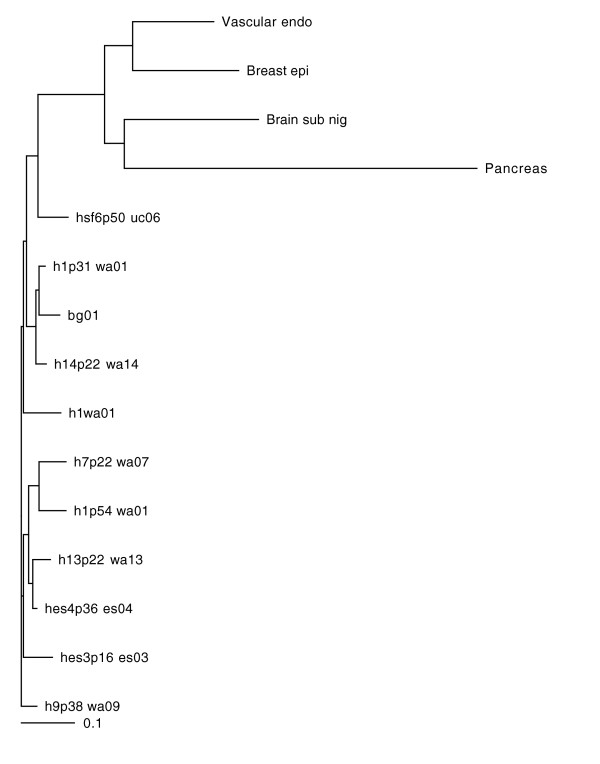
Pearson distance tree of human ESC libraries. ESC libraries do not cluster based on the genotype (compare WA01 and WA01-M), MEF feeder line (ES03 and ES04) or passage number (compare WA07 and WA01). Brain sub nig, LSAGE_Brain_normal_substantia_nigra_B_1; Breast epi, LSAGE_Breast_normal_myoepithelium_AP_IDC7; Pancreas, LSAGE_Pancreas_normal_B_1; Vascular endo; LSAGE_Vascular_endothelium_normal_liver_associated_AP_NLEC1.

To investigate the similarities and differences between the libraries, we performed hierarchical clustering using Pearson correlation coefficients [31]. For this comparison, we included data from four LongSAGE libraries generated from terminally differentiated cells (available from the Cancer Genome Anatomy Project [32]) to provide an 'out-group'. Figure 1 shows that the libraries for all nine human ESC lines form a cluster distinct from the libraries for the four terminally differentiated cell preparations, as expected. The ESC libraries also do not cluster together based on obvious commonalities between the lines, such as the MEF feeder lines used, sex chromosome karyotype or passage number.

**Figure 2:**
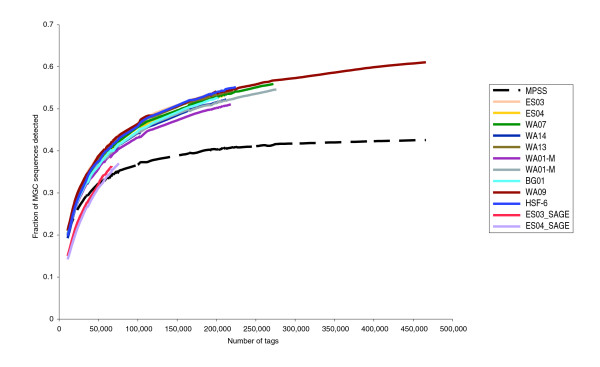
Coverage of the MGC by ESC sequence tag-based transcriptomes. Unambiguous tags from published MPSS and short SAGE ESC transcriptomes were mapped to genes in the MGC and compared to identically treated LongSAGE transcriptomes. To assess the impact of tag number on gene identification, the proportion of MGC sequences detected was plotted against increasing numbers of tags. Coverage of the MGC increases with increased numbers of tags for ESC LongSAGE libraries even at levels above 200,000 tags. In contrast, coverage of the MGC by the MPSS library plateaus early with little increase in coverage observed with increased sampling depth (MPSS). Coverage of the MGC by the short SAGE ESC libraries (ES03_SAGE and ES04_SAGE) is significantly lower due to the presence of ambiguous tags.

To assess the representation of known genes in the nine human ESC transcriptomes, we compared our data to other human sequence tag-based resources [15-18]. Highly expressed genes in each of the human ESC libraries showed significant overlap with previously published ESC SAGE [15] and MPSS datasets [18], but the diversity of genes identified by our LongSAGE data was significantly greater (Figure 2). To explore the functions encoded by transcripts detected in the LongSAGE libraries, we divided the genes (identified by uniquely mapping tags) into their respective Gene Ontology (GO) slim categories [33]. Pair-wise comparisons of individual human ESC libraries showed little difference in the relative proportions of each of the GO slim categories (Additional data file 3). In contrast, a similar comparison of individual or pooled ESC libraries to the differentiated cell lines showed a statistically significant increase, in the ESC libraries, in the proportion of transcripts encoding RNA binding proteins and mitochondrial proteins (*P *= 1.8 × 10^-7 ^and 1.0 × 10^-6^, respectively, by one-sided *t*-tests).

**Figure 3:**
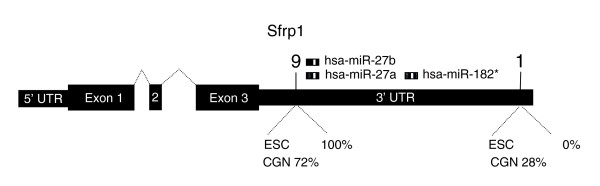
Differentially expressed isoforms (as predicted by LongSAGE tag positions) for the *Srfp1 *transcript (see text). The tag sequence at position 9 results in the loss of the 3' UTR region targeted by evolutionarily conserved miRNAs. Putative miRNA target sites were predicted using miRanda [34] and are represented by hashed boxes.

To investigate the potential functional significance of increased expression of transcripts for RNA binding proteins, we compared the global splicing profile of the ESCs and the four libraries of terminally differentiated cells. This was done by performing pair-wise comparisons across all transcripts in both the ESC and the terminally differentiated cell meta-libraries with the position of each uniquely mapped LongSAGE tag for which a transcript was known. These analyses did not reveal any difference in global transcript splicing patterns between the two meta-libraries, although differences in the relative abundance of specific transcript isoforms were identified. Of a total of 70 transcript isoforms found to be differentially expressed between the ESC and differentiated cell meta-libraries, 8 demonstrated statistical significance (*P *< 3.0 × 10^-5^; Additional data file 4). The most significantly affected transcript (lowest *P *value) encoded Secreted frizzled-related protein-1 (Sfrp1), a well characterized antagonist of WNT signaling. Our analysis suggested that the two isoforms of Sfrp1 we identified either retained or lost the 3' untranslated region (UTR; Figure 3). Only the transcript isoform lacking the 3' UTR was found exclusively in the ESCs. Closer examination of the 3' UTR region revealed putative miRNA target sites for two evolutionarily conserved miRNAs [34], the mouse homologues of which were found previously to be expressed in murine ESCs [35] (Figure 3). Given that activation of the canonical WNT signaling pathway induces differentiation and cell proliferation [36], we speculate that the expression of Sfrp1 may be regulated through miRNA-directed translational repression and that this regulation is bypassed through alternative 3' end formation in pluripotent ESCs.

We next examined the expression of transcripts that encode previously identified markers of undifferentiated ESCs. These include transcription factors such as Oct 4 [37], Nanog [4,5], the cell surface proteins tdgf-1 [38] and thy-1 [39], Lck [13], connexin cx43 [40], Rex1 [41] and Lefty-A and Lefty-B [42]. In addition, we looked for transcripts from six genes associated with early stages of ESC differentiation [14]. Table 2 shows the normalized gene expression levels across all cell lines. A similar pattern of expression is observed across all lines, with the exception of HSF-6, which exhibited a decrease in expression of ESC marker genes and a concomitant increase in expression of genes associated with differentiation, including alpha-fetoprotein. Notably, expression of Nanog, a divergent homeodomain protein that directs propagation of undifferentiated mouse ESCs [5], was not detected in the HSF-6 library. These features are consistent with the closer relationship of the HSF-6 library to libraries from differentiated tissues than to other ESC libraries (Figure 1). We therefore excluded the HSF-6 library from further analysis.

**Table 2:** Expression of undifferentiated and differentiated ESC markers

	Meta-libraries			Embryonic stem cell libraries									
		
Markers	CG-Meta	CGN-Meta	ESC-Meta	WA01	WA01-M	WA07	WA09	WA13	WA14	HSF-6	ES03	ES04	BG01
**Undiff**													
cx43	390.379	799.779	1635.9	1806.3	1182.32	1501.08	1155.02	1791.03	1720.1	970.938	2127.51	1486.37	2538.6
Oct4	0	0	1093.07	1433.46	1397.71	634.932	883.381	972.402	1376.08	432.023	1280.41	1247.4	1665.95
Tdgf1	13.9733	0	665.447	716.729	1131.92	767.057	252.394	339.21	801.146	329.585	374.871	578.3	1199.88
Sox2	43.6666	42.8454	406.121	398.183	265.793	352.332	675.905	257.8	518.389	320.677	350.528	358.451	416.489
Dppa4	11.3533	4.76059	386.637	463.34	595.745	572.54	290.895	303.028	494.826	231.6	189.87	430.141	421.447
Lefty-b	0	14.2818	245.218	173.753	9.16532	77.0727	32.0841	90.456	348.734	102.438	73.0268	258.084	233.036
Rex-1	38.4266	14.2818	105.813	112.215	82.4879	80.7428	132.614	31.6596	98.965	40.0846	160.659	62.1314	158.662
Lefty-a	0.873332	4.76059	102.454	54.2976	9.16532	25.6909	0	31.6596	70.6894	22.2692	24.3423	76.4694	168.579
Nanog	0	0	89.6892	65.1572	77.9051	110.104	66.3071	135.684	70.6894	0	92.5006	195.953	44.6238
Nodal	0	0	47.6999	21.719	4.58266	40.3714	6.41681	58.7964	84.8272	80.1692	48.6846	52.5727	64.4566
Foxd3	1.74666	0	23.1781	28.9587	45.8266	11.0104	32.0841	13.5684	9.42524	4.45385	24.3422	19.1174	49.582
Dppa2	0	0	14.7803	18.0992	36.6613	14.6805	4.27787	0	9.42525	4.45385	4.86845	14.338	69.4148
Lck	0	0	13.1007	14.4794	13.748	18.3506	0	22.614	23.5631	4.45385	4.86845	23.8967	14.8746
utf-1	0	0	11.0852	10.8595	18.3306	0	10.6947	13.5684	0	17.8154	14.6054	0	34.7074
Tert-1	0	0	6.7183	10.8595	13.748	7.34026	10.6947	9.0456	4.71262	4.45385	4.86845	0	0
abcg2	0.873332	14.2818	2.01549	0	4.58266	3.67013	0	0	0	4.45385	4.86845	0	4.9582
dppa3	0	0	1.34366	0	4.58266	0	2.13894	0	0	4.45385	4.86845	0	4.9582
cx45	0	0	0.67183	0	4.58266	3.67013	0	0	0	0	0	0	0
													
**Diff**													
brachyury	0	0	3.35915	0	0	0	0	40.7052	0	4.45385	0	0	0
afp	0	0	8.06196	3.61984	0	0	8.55575	49.7508	4.71262	334.038	9.7369	4.77934	4.9582
krt15	52.3999	0	0.335915	0	4.58266	0	0	0	0	0	0	0	0
sox-1	8.73332	0	0.335915	0	0	0	0	0	0	8.90769	0	0	0
fgf5-1	48.0332	14.2818	22.1704	21.719	27.496	11.0104	10.6947	13.5684	18.8505	17.8154	19.4738	28.676	19.8328

Expression of defined ESC markers normalized to tags per million. Data for Meta ESCs (Meta-ESC), malignant (Meta-CG) and normal differentiated (Meta-CGN) cells are included for comparison. Diff, differentiated; undiff, undifferentiated.

A previous analysis of SAGE data generated using ES03 and ES04 cells showed that Rex1 was within the top 25 differentially expressed transcripts, with no Rex1 tags detected in the ES04 line and an absence of Rex1 expression in ES04 cells confirmed by quantitative and semi-quantitative real time (RT)-PCR [15]. Interestingly, in our LongSAGE libraries, tags for Rex1 were present in all nine ESC libraries, including the library prepared from ES04 cells and there was less than a three-fold difference in Rex1 expression between ES03 and ES04 (Table 2).

To generate a list of transcripts common to all libraries (excluding the HSF-6 library because of the differentiation markers found therein), we first identified tags from each library that uniquely mapped to transcripts within RefSeq [43] and the Mammalian Gene Collection (MGC) [44]. This analysis identified a set of 4,337 LongSAGE tags present in all libraries (Additional data file 5). Comparison of this list to those generated by previous MPSS and SAGE approaches revealed extensive (80%) concordance between the SAGE-based transcriptomes. In contrast, 52% of genes identified by MPSS were not found in either of the SAGE common gene lists. Some of this lack of concordance may be explained by differences in the tagging restriction enzyme used by the two protocols (*Nla*III for SAGE and *Dpn*1 for MPSS) and the fact that different mRNA preparations were used in each study. To further explore this lack of concordance, we compared the longSAGE and MPSS-derived gene lists to a common gene list derived from Affymetrix expression arrays generated from the same RNAs used to construct our LongSAGE libraries [45]. The Affymetrix common gene set contained more than 80% of the LongSAGE common gene list (Additional data file 5) while MPSS contained only 68% of the genes on this list.

### Identification of novel ESC-specific transcripts

LongSAGE offers opportunities for discovering novel transcripts. These can be identified as tags that map uniquely to the genome but not to any available transcript resources. To look for these, we used the 2.5 million tag meta-library, which contained 379,645 unique tag sequences. Grouping LongSAGE tags that mapped to genomic locations in close proximity to one another [30] resulted in the identification of 24,593 transcription units. Of these, 14,588 did not overlap with known genes and were classified as novel. Most tags were expressed at low levels with 46% (6,672) identified by a single LongSAGE tag. Even though singletons are enriched for artifacts, many of these are likely to represent real transcripts, for two reasons: first, they map to the genome; and second, we [30] and others [46] have shown previously that at least 70% of novel, singleton, high quality LongSAGE tags identify rare transcripts whose expression can be confirmed in RNA-dependent RT-PCR experiments.

To further characterize these putative novel, low-abundance ESC library specific transcripts, we compared the ESC meta-library to publicly available data derived from 247 non-ESC SAGE libraries that together contained 654,491 unique tag sequences. This comparison identified 20,047 tag sequences found only in the human ESC meta-library (Additional data file 6). For subsequent analyses, we focused on those tags that uniquely mapped at least 2 kb away from any known gene. This analysis reduced the number of tags to 634 (Additional data file 7), of which 301 were found within genomic regions exhibiting sequence conservation between human and mouse or rat (Additional data file 8). We used rapid amplification of cDNA ends (RACE) [47,48] to clone the 5' ends of 52 of these (Additional data file 9). Alignment of the resulting sequences to the human genome revealed that 22 (40%) were spliced. An open reading frame (ORF) scan of the 52 RACE clone sequences using Bioperl [49] tools and custom scripts identified 6 transcripts that encoded peptides longer than 100 amino acids in length. However, with the exception of one transcript (HA_003333) that overlapped the 3' end of the *MAPK2 *gene, none of the identified ORFs demonstrated Ka/Ks ratios suggestive of purifying selection [50]. Hence, these transcripts may not encode proteins but may instead represent non-coding RNAs (ncRNAs).

**Figure 4:**
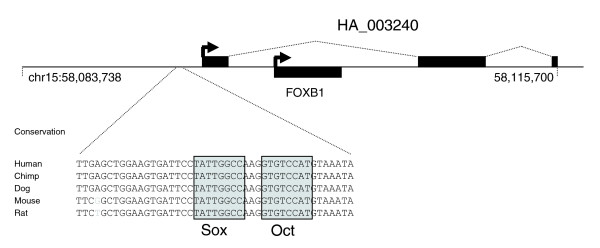
Structure of the HA_003240 transcript. Alignment of the 5' RACE sequence for HA_003240 to chromosome 15 sequences, showing its position relative to the nested single exon transcript *Foxb1 *and conservation of the Octamer/Sox binding elements within the promoter region.

Four RACE clones were found to have genomic coordinates that overlapped with those of known transcripts (Additional data file 9). One of these (HA_003240; Figure 4) is of particular interest because it contains the entire coding sequence of the *Foxb1 *gene within its first intron. *Foxb1 *encodes a winged helix transcription factor involved in the development of the vertebrate central nervous system and *Foxb1*^-/- ^mice display phenotypes consistent with a requirement for this gene in both embryonic and postnatal stages of development [51-53]. Interestingly, the ESC meta-library did not contain any tags corresponding to known *Foxb1 *transcripts except for a single *Foxb1 *tag in the HSF-6 library. This general lack of *Foxb1 *expression in ESCs and the genomic location of the *Foxb1 *gene within the first intron of HA_003240 are consistent with the notion that *Foxb1 *expression is repressed by expression of HA_003240, possibly by steric inhibition of the transcription initiation complex [54]. The HA_003240 sequence overlaps partially with an EST obtained from an undifferentiated human ESC line (CD049816), as well as with ESTs from an embryonic carcinoma line, a kidney carcinoma line and hypothalamus tissue (for example, DA713666, DB173211 and BI458015, respectively). Examination of the promoter region of HA_003240 revealed the presence of highly conserved sequences containing an Oct/Sox binding element, suggesting that HA_003240 expression may be maintained in pluripotent ESCs through the recruitment of an Oct4/Sox2 complex (Figure 4). *Oct4 *encodes a transcription factor that regulates a number of key human ESC markers, including Nanog, through co-operative binding with a Sox family member [55]. Given the documented role for Foxb1 in controlling the differentiation of neuronal cell types, the genomic organization of the *Foxb1 *locus is intriguing and suggests an interesting mechanism for negatively regulating *Foxb1 *expression in Oct4-expressing cells.

Many pseudogenes have been identified in the human genome using homology-based approaches [56-58]. Pseudogenes are generally not transcribed due to their lack of functional promoters [59,60]. However, there are examples of pseudogenes that have retained or acquired functional promoters, leading to their transcription [61]. Because of the low levels of expression of the 52 novel transcripts (on average, only 3 tags per million) we asked whether the 5' RACE clones were derived from expressed pseudogenes. Comparison of the RACE clone sequences to three computationally generated lists of known human pseudogenes [56-58] revealed only one clone (HA_003350) with a predicted pseudogene contained within its exon. Furthermore, with the exception of HA_003333, none of the novel transcript sequences showed significant sequence similarity to any known ORF (using a 70% ORF threshold [58]). Taken together, these analyses do not support the notion that the novel genes identified by our analysis are enriched for expressed pseudogenes.

**Figure 5:**
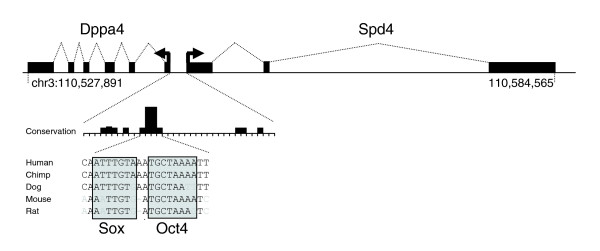
Structure of the *Spd4 *transcript. Alignment of the full-length *Spd4 *transcript on chromosome 3 showing its position relative to *Dppa4 *and conservation of the Octamer/Sox binding elements within the promoter region.

To more fully characterize a transcript identified by a singleton tag (Additional data file 9), we attempted to recover a full length transcript using 5' and 3' RACE and primers annealing within the terminal exon of the putative transcript. Alignment of the resulting candidate full length sequence to the human genome revealed a transcript that contained two introns (Figure 5). Examination of the genomic region surrounding this transcript showed that it resides in a region of the long arm of chromosome 3 (chr3:110,539,351-110,584,565) lacking annotated transcripts. The putative transcriptional start is located 266 bp from the transcriptional start site of *Dppa4*, a gene known to have an expression pattern in ESCs that is similar to that of *Oct4 *[62] (Figure 5). To investigate the possibility that this promoter region is regulated directly by Oct4, we looked for the presence of conserved Octamer and Sox (high mobility group (HMG)) elements. A single 20 bp region of cross-species sequence conservation was found that contains a consensus binding element for an Octamer/Sox dimmer, suggesting that the novel gene is regulated by Oct4/Sox2 (Figure 5; chr3: 110,539,180-111,539,200). In support of this finding, the conserved region was found to reside within a probe identified by chromatin immunoprecipitation (ChIP)/CHIP [63] as a target of Oct4 and Sox2 (Probe spans chr3: 110,539,028-110,539,588). Taken together, these analyses suggest that both *Dppa4 *and the novel transcript are divergently transcribed from a common promoter bound by an Oct4/Sox complex. Based on its proximity to the *Dppa4 *gene we have named this novel transcript *Spd4 *(for 'shares promoter with *Dppa4*').

Comparison of the 5' RACE clone sequences to publicly available ESTs revealed 36 (69%) with matches to other ESTs, of which 7 were found only in data derived from pluripotent human ESC lines. One RACE clone that overlapped an EST derived from pluripotent human ESC lines (HA_003152) was also found to be expressed in all nine ESC lines studied here. BLAT [64] alignment of the 5' RACE clone sequence to the human reference genome sequence revealed that HA_003152 contained two introns and resided within a genomic region that exhibited sequence similarity to long interspersed nuclear elements. An ORF scan revealed a 129 amino acid peptide encoded in the second exon with homology to the carboxyl terminus of the LINE *p40 *ORF.

**Figure 6:**
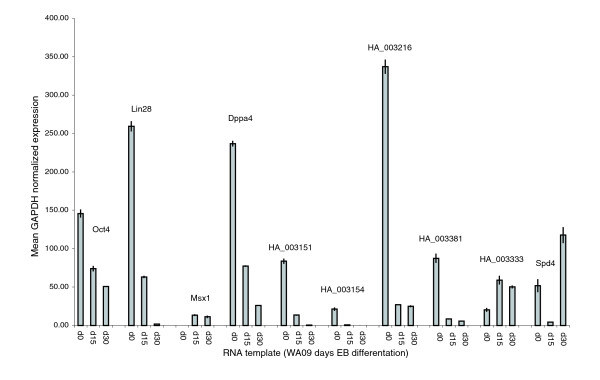
Expression of selected transcripts during embryoid body differentiation. qPCR was used to monitor expression of selected transcripts in ESCs stimulated to differentiate into embryoid bodies. Three control markers, Oct4, Lin28 and Msx1, were included. Expression levels are reported as the mean of triplicate measurements and are normalized to GAPDH.

To explore the expression pattern of the HA_003152 transcript we used quantitative RT-PCR (qPCR) to compare transcript levels in RNA purified from human ESCs maintained under conditions that promote their maintenance in an undifferentiated state to RNA extracts obtained from human ESCs that had been stimulated to differentiate into embryoid bodies. To provide a comparative dataset we selected five additional novel transcripts for qPCR. In all cases, qPCR amplicons were designed to cross exon-exon boundaries. As controls we also monitored expression of *Oct4*, *Lin28 *and *Msx1 *in the same RNA preparations. Figure 6 shows the expected expression pattern for the control gene set, with a reduction in expression of *Oct4 *and *Lin28 *in the human ESCs stimulated to differentiate into embryoid bodies and an up-regulation of expression of the early differentiation marker *Msx1*. Significant reduction of expression was observed in four of the six transcripts tested, including HA_003152, whose expression was undetectable at d30 (Figure 6). These transcripts are hence potential markers of pluripotency.

## Conclusion

As part of the ongoing effort to elucidate mechanisms regulating ESC self-renewal, we generated 2.5 million LongSAGE tags from nine human ESC lines. Comparison of these data to libraries prepared from differentiated tissues identified a group of ESC-library specific transcripts and an enrichment of transcripts encoding mitochondrial and RNA binding proteins (by comparison to differentiated cells). RNA binding proteins play a role in the regulation of mRNA processing and examination of non-canonical longSAGE tags in the human ESC libraries suggest that these cells express a distinct collection of gene isoforms. One such isoform may bypass translational down regulation through the expression of a transcript lacking predicted miRNA target sequences.

An emerging theme in digital gene expression profiling is the identification of a large class of transcripts that map uniquely to the genome, but cannot be localized to any known or computationally predicted transcripts. Tags in this class are predominantly found at relatively low levels. Analysis of the 2.5 million LongSAGE tags generated in the course of this study revealed 14,588 such tag sequences, a subset of which were found exclusively in human ESCs. As a first step towards understanding the relevance of these transcripts to ESC biology we generated 5' RACE clones for 52 novel apparently ESC-specific transcripts. Analyses of these transcripts revealed that the majority do not appear to encode proteins and do not overlap existing pseudogene predictions. One transcript was found to be expressed across all nine ESC lines we profiled and matched ESTs generated by others from ESCs. Its restricted expression pattern suggests that it may represent a novel transcriptional marker for the maintenance of pluripotentiality. In addition to the discovery of this potential marker, we also identified four novel transcripts that may participate in the regulation of expression of known genes, one of which is known to play a direct role in differentiation. Our analyses indicate that there are many previously undiscovered transcripts expressed in human ESCs and support the contention that sampling of SAGE libraries to depths beyond currently accepted practice is required to fully explore the coding potential of the mammalian transcriptome. To assess possible functions associated with such rare transcripts, we are actively pursuing the cloning and characterization of the remaining novel human ESC-specific transcripts identified in this study.

## Materials and methods

### Cell culture and RNA isolation

Detailed information regarding the human ESC lines used in this study can be found at the NIH Stem Cell Information website [65]. The passage numbers of the cells analyzed in this study are presented in Table 1. Total RNA was prepared using Trizol reagent (Invitrogen, Burlington, ON, USA) following the manufacturer's protocol and was assayed for quality and quantified using an Agilent 2100 Bioanalyzer (Agilent Technologies) and RNA 6000 Nano LabChip kit (Caliper Technologies, Hopkinton, MA, USA).

### LongSAGE library construction

Nine LongSAGE [29] libraries were constructed from 5-20 μg of DNase I-treated total RNA as described [30] (DNase I from Invitrogen). LongSAGE data generated for this study are available through our embryonic stem cell transcriptomes website [45] and through the CGAP web portal [32].

### Novel transcript identification

LongSAGE tags of at least 99.9% accuracy (calculated using Phred [66,67] quality scores) from the meta-library were compared to 247 publicly available human SAGE libraries (GEO [68], Discovery db [69]). To allow direct comparison of the LongSAGE data to the 14 bp SAGE tags available in the public libraries, the 3' ends of the 21 bp tags were truncated *in silico *to form 14 bp tags. A total of 2,508,608 tags corresponding to 222,337 unique 14 bp tag sequences (379,465; 21 bp parental sequences) were utilized in this analysis. These tags were directly compared to all unique tags from the human SAGE libraries to generate a list of tags found solely in the ESC meta-library.

Tag-to-gene mapping was performed using the comprehensive mapping of SAGE tags (CMOST) software [69] as follows. Tags were mapped to various publicly available transcript databases in a hierarchical fashion with the highest quality transcript databases used first. As tags were mapped to a known transcript in a higher quality database, they were excluded from further analysis with subsequent lower quality databases to mitigate redundancies arising from lower quality DNA sequence resources. The following databases were used for CMOST tag-to-gene mapping in this order: MGC [70], RefSeq [71], Ensembl transcripts [72] (exon sequences only), Genbank Human Mitochondrial Sequence (accession AY289102.1), Genbank Non-coding sequences [73], Ensembl genes [72] (1,000 bp UTR and intron sequences included), Ensembl ESTs [72], and Golden path genomic contigs (Genbank Human Genome Assembly Contigs build 34, January 2004 [73]). In addition to allowing perfect matches, the CMOST approach attempts to account for single base permutations, insertions and deletions, improving the rate of tag-to-gene mapping.

### SAGE tag-to-gene mapping

LongSAGE tags were mapped to known and computationally predicted transcripts using versions of the following databases available as of March, 2005: RefSeq [71], RefSeqX [71], Mammalian Gene Collection [70], and RefSeqGS [71]. Tags were also mapped to human genomic sequence using the NCBI Reference Sequence Genome database [71], release 35, August 2004. From the genome sequence, a table was generated containing all 27.4 million potential SAGE tags adjacent to genomic *Nla*III restriction sites (CATG). Of these, our analysis defined a subset of 19.4 million genomic tag sequences that were unique within the genome.

A second table was generated that stored information about exons: genome sequence contig, transcript orientation, exon number, exon boundary type and nucleotide positions of exon boundaries for all approximately 267,000 exons annotated on release 35 of the Reference Sequence genome. The LongSAGE tag sequences were compared to the unique genomic tag table, yielding sets of genomic positions for all tags in the library. These in turn were compared to the table of exon information, producing a mapping for each tag relative to annotated exons.

### Statistical analysis

For the GO category comparisons, a standard *t*-test comparing two samples was used. The null hypothesis was that the two samples arose from populations with the same mean and standard deviation. The values within each sample were the number of GO categories represented in each library of the set, nine in the ESC set and four in the normal set. To account for variation due to library size, only the transcripts with the top 1,000 expression values were included. A one-sided *p *value was reported. Microsoft Excel was used to perform the computation.

To select differentially expressed LongSAGE tags, the ESC and CGN meta-libraries were compared on a tag per tag basis to obtain a *p *value for the null hypothesis that the two tag frequencies arose from Poisson distributions with the same mean. This was derived using a normal approximation to the Poisson as described by Kal *et al*. [74]. All transcripts that showed differences with a significance of *p *< 0.05 were selected. Tag counts were converted to tags per million, and transcripts that differed by less than three-fold were eliminated. All pairs of tags existing within the same transcript were then listed if the differential expression for the two tags was in the opposite direction.

### RACE

First strand 5' and 3' RACE ready cDNA was synthesized from 2.0 μg of DNase I (DNA-*free*™ kit; Ambion, Austin, TX, USA) treated RNA using the BD SMART RACE cDNA Amplification kit following the manufacturer's recommended protocol (BD Biosciences Clontech, Mountain View, CA, USA). Gene specific 5' RACE primers were designed using custom scripts and Primer 3 [75] to lie downstream of the target LongSAGE tag with an optimal Tm of 68°C (Additional data file 10). For 3' RACE reactions a series of primers were designed manually based on the 5' RACE clone sequence (Additional data file 10). The cDNA was amplified using the Phusion™ High-Fidelity PCR Kit (MJ Research, Inc., Waltham, MA, USA) following the manufacturer's recommended protocol with the addition of DMSO to a final concentration of 3%. The cycling conditions consisted of an initial denaturation at 98°C for 30 seconds followed by 10 touchdown PCR cycles starting with 98°C for 10 seconds, 72°C (decreased by 1°C in each subsequent cycle) for 15 seconds, 72°C for 30 seconds; then 29 cycles of 98°C for 10 seconds, 62°C for 15 seconds, 72°C for 30 seconds; followed by an extension at 72°C for 10 minutes. PCR product for each sample (10 μl) was loaded on a 1.2% agarose gel and subjected to electrophoresis for 3.5 hours at 110 mA in 1× TBE buffer (Tris/Boric Acid/EDTA). The gel was stained with SYBR Green (Mandel, Guelph, ON, Canada) and visualized using a Typhoon 9400 Variable Mode Imager (Amersham, Baie d'Urfe, PQ, Canada). Amplicons were extracted from the gel, purified and cloned into the pCR4^®^-TOPO^® ^vector using the TOPO TA Cloning^® ^Kit for Sequencing (Invitrogen). Plasmid vectors were electroporated into bacterial cells, and recombinant clones were selected on agar plates containing appropriate antibiotics as described [76]. Glycerol stocks were prepared from 12 individual clone isolates per amplicon and stored in 384-well plates. Clone inserts were sequenced on an ABI PRISM 3730 XL DNA Analyzer using BigDye primer cycle sequencing reagents (Applied Biosystems, Foster City, CA, USA).

### Quantitative RT-PCR

RNA was obtained from H9 cells before and after induction of differentiation using a 30-day embryoid body protocol. Undifferentiated H9 cells maintained for 7 days on matrigel (BD Biosciences, San Jose, CA, USA) in media conditioned by mouse embryonic fibroblasts and supplemented with 4 ng/ml fibroblast growth factor (bFGF-2) were harvested for embryoid body formation. Briefly, the cells were incubated with TrypLE (Invitrogen) for 10 minutes at 37°C and then collected by scraping. Resultant cell aggregates were subsequently cultured in non-adherent dishes using KOSR-based media without FGF2, for 15 to 30 days. At appropriate time-points RNA was extracted into Trizol. cDNA was synthesized from 2.0 ug of DNase I (DNA-*free*™ kit, Ambion) treated total RNA using the SuperScript Choice System following the manufacturer's recommended protocol (Invitrogen). Gene specific primer pairs were designed using custom scripts and Primer 3 [75] to amplify approximately 150 bp of the target gene with an optimal Tm of 68°C (Additional data file 10). Whenever possible amplicons were designed to cross exon/intron boundaries. Amplification was performed in a 10 μl reaction mixture containing 5 μl of 2× SYBR Green PCR Master Mix (Applied Biosystems), 2 μl of template cDNA, and 250 pmol of the forward and reverse primer pair. After preparation of the reaction mixtures in 96-well plates, the plates were centrifuged at 800 rpm for 1 minute in an Eppendorf 5810 swing rotor centrifuge (Eppendorf, Westbury, NY, USA). Amplification and detection were performed on an ABI Prism 7600 Sequence Detection System (Applied Biosystems). The PCR protocol consisted of the following: a single cycle of 10 minute at 95°C and 40 two-step cycles, with one cycle consisting of 15 seconds at 95°C and 60 seconds at 60°C. Results were analyzed as described [77] using a GAPDH probe for normalization.

## Additional data files

The following additional data are available with the online version of this paper. Additional data file 1 is a summary of mouse specific tag types identified. Additional data file 2 is a table of genomic mappings for 268,515 unique tag sequences found in nine independent human embryonic stem cell lines. Additional data file 3 is a Gene Ontology analysis of nine independent human embryonic stem cells. Tag counts are expressed for each GO category for the top 1,000 by tag count. Additional data file 4 lists statistically significant differentially expressed LongSAGE tags found between embryonic stem cells and terminally differentiated tissues. Additional data file 5 is a table listing the 4,337 genes found in common across 8 undifferentiated human embryonic stem cell lines. Additional data file 6 is a table listing the 20,047 LongSAGE tags exclusively expressed in embryonic stem cell lines. Additional data file 7 is a table listing the 634 LongSAGE tags exclusively expressed in ESCs that uniquely map to the human genome at least 2 kb away from an annotated transcript. Additional data file 8 is a table listing the 301 LongSAGE tags exclusively expressed in ESCs that uniquely map to species conserved regions of the human genome at least 2 kb away from an annotated transcript. Additional data file 9 is a table listing the 52 ESC specific transcripts identified by 5' RACE. Additional data file 10 lists the RACE and qPCR primer sequences used in this study.

## Supplementary Material

Additional data file 1 Summary of mouse specific tag types identified. Click here for file

Additional data file 2 Genomic mappings for 268,515 unique tag sequences found in nine independent human embryonic stem cell lines. Click here for file

Additional data file 3 Tag counts are expressed for each GO category for the top 1,000 by tag count. Click here for file

Additional data file 4 Statistically significant differentially expressed LongSAGE tags found between embryonic stem cells and terminally differentiated tissues. Click here for file

Additional data file 5 The 4,337 genes found in common across 8 undifferentiated human embryonic stem cell lines. Click here for file

Additional data file 6 The 20,047 LongSAGE tags exclusively expressed in embryonic stem cell lines. Click here for file

Additional data file 7 The 634 LongSAGE tags exclusively expressed in ESCs that uniquely map to the human genome at least 2 kb away from an annotated transcript. Click here for file

Additional data file 8 The 301 LongSAGE tags exclusively expressed in ESCs that uniquely map to species conserved regions of the human genome at least 2 kb away from an annotated transcript. Click here for file

Additional data file 9 The 52 ESC specific transcripts identified by 5' RACE. Click here for file

Additional data file 10 RACE and qPCR primer sequences used in this study. Click here for file
